# Human resource time commitments and associated costs of Community Caregiver outreach team operations in South Africa

**DOI:** 10.1371/journal.pone.0282425

**Published:** 2023-03-06

**Authors:** Rachel Mukora, Ryan R. Thompson, Piotr Hippner, Resignation Pelusa, Martha Mothibi, Richard Lessells, Alison D. Grant, Katherine Fielding, Kavindhran Velen, Salome Charalambous, David W. Dowdy, Hojoon Sohn

**Affiliations:** 1 The Aurum Institute, Aurum House, Johannesburg, South Africa; 2 The School of Public Health, University of the Witwatersrand, Johannesburg, South Africa; 3 Department of Epidemiology, Johns Hopkins Bloomberg School of Public Health, Baltimore, MD, United States of America; 4 Africa Health Research Institute, KwaZulu-Natal, South Africa; 5 TB Centre, London School of Hygiene & Tropical Medicine, London, United Kingdom; 6 Department of Preventive Medicine, Seoul National University College of Medicine, Seoul, South Korea; Universidad Nacional Autonoma de Nicaragua Leon, NICARAGUA

## Abstract

**Introduction:**

In South Africa, Community Caregivers (CCGs) visit households to provide basic healthcare services including those for tuberculosis and HIV. However, CCG workloads, costs, and time burden are largely unknown. Our objective was to assess the workloads and operational costs for CCG teams operating in different settings in South Africa.

**Methods:**

Between March and October 2018, we collected standardized self-reported activity time forms from 11 CCG pairs working at two public health clinics in Ekurhuleni district, South Africa. CCG workloads were assessed based on activity unit times, per-household visit time, and mean daily number of successful household visits. Using activity-based times and CCG operating cost data, we assessed CCG annual and per-household visit costs (USD 2019) from the health system perspective.

**Results:**

CCGs in clinic 1 (peri-urban, 7 CCG pairs) and 2 (urban, informal settlement; 4 CCG pairs) served an area of 3.1 km^2^ and 0.6 km^2^ with 8,035 and 5,200 registered households, respectively. CCG pairs spent a median 236 minutes per day conducting field activities at clinic 1 versus 235 minutes at clinic 2. CCG pairs at clinic 1 spent 49.5% of this time at households (versus traveling), compared to 35.0% at clinic 2. On average, CCG pairs successfully visited 9.5 vs 6.7 households per day for clinics 1 and 2, respectively. At clinic 1, 2.7% of household visits were unsuccessful, versus 28.5% at clinic 2. Total annual operating costs were higher in clinic 1 ($71,780 vs $49,097) but cost per successful visit was lower ($3.58) than clinic 2 ($5.85).

**Conclusions:**

CCG home visits were more frequent, successful, and less costly in clinic 1, which served a larger and more formalized settlement. The variability in workload and cost observed across pairs and clinics suggests that circumstantial factors and CCG needs must be carefully assessed for optimized CCG outreach operations.

## Introduction

In low- and middle-income countries (LMICs), Community Health Workers (CHWs) are a cadre of healthcare workers that can help alleviate shortages of healthcare staff and achieve the goals of the primary healthcare system [1]. CHWs can be found in many regions of the world, from Ethiopia to Indonesia [2]. However, CHW programmes differ in their design, including the type of worker, level of training, scope of work, nature of supervision and the extent to which basic equipment is provided [2]. In South Africa (SA), Community Caregiver (CCG) outreach teams, also previously known as Ward-Based Outreach Teams (WBOTs), were established in 2011 as part of the Re-engineering Primary Health Care (RPHC) reform [3]. The goal of the CCG initiative was to support the delivery of primary healthcare (PHC) services in South Africa. The CCG teams bridged the gap between households and health care facilities, strengthening preventive healthcare within communities [3]. The CCG initiative brought services to patient homes without requiring a clinic visit, reducing the time and financial burdens on households to receive routine care. CCGs were conceptualised as an extension of existing primary healthcare (PHC) facilities, with facility managers providing the oversight, support and supervision of teams [4].

CCG outreach teams are normally comprised of about five CCGs and a team leader, typically a professional nurse [5]. Each CCG team is assigned a municipal ward where they provide health services to the community such as HIV, tuberculosis (TB), maternal and child health, and chronic condition care through household visits, typically working in pairs of 2–3 individual CCGs [3]. However, there are challenges in operating community-oriented care programs such as CCGs, both in South Africa and elsewhere in Sub-Saharan Africa [6, 7]. In South Africa, CCG coverage is unevenly distributed, with complete coverage in only half of the country’s 4,277 wards [8]. In March 2017, there were 3,275 CCGs teams—representing only 42% of the estimated 7,800 teams required to provide comprehensive national care [4]. And, while CCGs do ease access to care in communities, they have limited understanding of certain interventions, can overemphasize care for certain diseases and strategies, have high workloads, and can experience pushback from communities based on stigma and perception [7, 9–11].

Community-based care interventions like CCGs are only implementable with an appropriate understanding of resource requirements [7, 9–11]. Ideally, these programs aim to maximize the number of successful visits on a per day basis, since the number of households covered by each CHW is based on distance and travel time between households, demographic structure, and burden of disease [4]. Yet, lack of understanding by management may affect the quality of support and supervision of the CCG teams [4, 9, 12]. The impact of these challenges to the healthcare sector include inefficient use of labor and financial resources for health, such that public health service delivery does not result in optimal healthcare outcomes [13, 14].

There is growing evidence demonstrating the value and contribution of CCG teams in improving gaps of community health programs in South Africa. However, few economic analyses have evaluated both the operationalization and costs of CCG outreach programs to inform evidence-based resource allocation. Uncertainty in human resource and financial requirements can lead to CCG programs that do not make the best use of available resources. The primary objective of this manuscript was to evaluate the human resource and financial requirements necessary for implementation of CCGs in diverse settings. In this study, we assessed workloads and operational costs of CCG teams operating from two public health clinics in Ekurhuleni district. We report differences in CCG operations, human resource needs in delivering CCG-based care, and activity-based costs of services provided by the CCG teams.

## Materials and methods

### Overview of parent study, Asibambisane

The Asibambisane (“let’s work together”) study was the first project to formally evaluate the role of CCG teams in TB household contact tracing. The study took place in three high TB and HIV burden districts in South Arica: Ekurhuleni, an urban district (Gauteng province); Bojanala, a semi-urban district (North West province); and uMkhanyakude, a rural district (KwaZulu-Natal province). None were National Health Insurance pilot districts. Study clinics implemented an improved CCG model to improve quality of contact tracing by using an app to help CCGs document their activities and hiring a quality improvement coach to visit study clinics and provide performance feedback and advice to CCG teams [9]. An optimized CHW strategy was implemented that included: (1) improved training focused on TB case finding and contact tracing, (2) improved monitoring and evaluation by implementation of an electronic hand-held device, (3) improved integration with the facility by introducing new tools and an improved understanding of issues encountered, (4) improved supervision of the CHW’s through enhanced monitoring and support of the team leaders [9, 15].

The Asibambisane training programs involved integrated training where CHW’s received training prior to deployment in the community, which covered a wide range of topics related to their role. Unlike typical CCG training programs, which have minimal in-service training once CHW’s are operational, Asibambisane tested an integrated training approach, where CHW’s, supervisors, and team leaders were trained together with facility-based TB focal nurses and TB programme personnel. This involved regular on-site group trainings.

### Site and participant selection

The time and motion (TAM) and costing studies took place in two primary healthcare clinics (PHCs) in Ekurhuleni district, Gauteng. The clinics were selected because they represented distinct contexts. One site was based close to a farming area (clinic 1), whereas the second site was based close to an informal urban settlement (clinic 2). All CCGs were invited to informational meetings where we described the study purpose, and those interested in participating were recruited for TAM data collection. Between March 2018 and October 2018, we recruited 11 CCG pairs working as members of CCG teams to evaluate activity-based time commitments of CCG operations.

### Data collection

#### Time and motion

The TAM assessment focused on all field-based activities conducted by the CCG pairs from the time they left the clinic for household visits to the time they returned to the clinic. Our study team developed a standardized time reporting form based on reviews of CCG activity reports, discussions with CCGs, and direct on-site observations during a preceding pilot phase (January to March 2018). All participating CCG staff were trained to self-report TAM data during the study period. During each observation day, one member of the CCG pair recorded start and end times for each discrete activity (categorized based on a pre-defined set of activity codes shown in Table S1.1 in S1 Appendix) carried out by their peer in the field [16, 17]. Times and activities were recorded in a continuous and consecutive manner with no time gaps between activities [17].

TAM data were collected in two distinct ways: at the household level and at the daily level. Household visit TAM forms captured the type and duration of activities performed within each household visit. Daily TAM forms captured information about the total time spent performing field work each day, the number of households visited, how much time was spent at patient households, how much time was spent traveling, and reasons for unsuccessful household visits. Both TAM forms were completed concurrently with each other, so data between forms could be linked. Direct activities were those that involved CCG service provision at households, namely household registrations, follow-up visits and other contact investigations (for TB and other diseases, such as maternal and child health and HIV). Indirect activities included travel from the clinic to the household, from one household to another, and back to the clinic.

Each CCG pair was asked to complete both TAM forms at least three working days per calendar month during the study period, for an anticipated 264 form submissions. All submissions were voluntary and CCG pairs were not given specific days to collect data (i.e. submission and date selection was random). Completed paper-based forms were collected weekly by two trained study research assistants for data validation and were recorded into a Microsoft Excel database. The data validation process involved assessing the quality of each TAM form, based on the completeness and adherence to the time and activity reporting guidelines provided, with poor-quality forms excluded. Poor-quality forms were defined as forms that were not informative for TAM activities, such as forms with time gaps or missing information and those with suspicious patterns like all activities lasting for an equal duration. Feedback and quality reports were given to all CCG pairs to help improve quality of data collection over the study duration. Any data discrepancies were resolved by two independent reviewers reviewing the original data. Person-time–both in total and broken down per day, household visit, and patient interaction–were calculated and assessed for both clinics separately.

#### Costs

We assessed costs from a health system perspective. The unit prices of medical equipment and general consumables were taken from market prices at the time of purchase [18]. Annual overhead and building costs of the CCG operations were taken from financial records kept at the supporting clinics. Salaries of personnel involved in the program were collated from a publicly available salary portal [19]. Implementation costs related to preparation for launching the CCG intervention were tracked by the central study team and included activities like training CCG teams and developing training materials.

### Analysis

#### Time and motion

To assess the CCG outreach teams’ workloads, we evaluated a number of outcomes based on our TAM data: 1) total duration of a typical workday in carrying out household visits (assessing proportion of time spent travelling between households and median duration of a household visit), 2) median unit time for key activities carried out by CCG pairs in their typical workday (including within-household activities), 3) composition of activities carried out by each type/purpose of household visit, 4) frequency and reasons for unsuccessful household visit attempts (defined as a failure to interact with patients at a household), 5) changes in the frequency and duration activities were performed over time, and 6) the proportion of household visits that involved TB-related care (contact tracing, treatment adherence counselling, etc). The unit for all TAM analyses was the CCG pair.

Results are presented by clinic, with information by CCG pair available in the appendix. Summary statistics were used to describe TAM outcomes of interest. Trends in frequency and duration of different activities over time were assessed by grouping TAM data into bi-monthly bins.

#### Costing

The cost analysis was done using both bottom-up and top-down approaches via a costing model developed in Microsoft Excel (v15.26, 2016, Microsoft Corp., Redmond WA). We depreciated capital goods linearly over their expected life-years, which we assumed to be between two and five years. Thus, we took the annual cost of capital goods to be equal to their purchase price, divided by these expected life-years. In addition to this linear depreciation, we also discounted all future values at 5% per year. All costs were presented in 2019 US dollars (USD) using the World Bank exchange rate of 1 USD = 14.448 South African Rand (ZAR) [20].

Using a top-down approach, we estimated overall cost per minute by dividing the total cost estimates for each cost category by annual PHC operation time, assuming 250 working days per year and 8 working hours per day. The per-minute cost estimates were then merged with the TAM data to develop estimates of the cost per activity and per household visit, based on their mean/median durations.

Using a bottom-up approach, we estimated a cost per minute estimate at both clinics for each cost category by apportioning costs to discrete CCG pairs and activities based on their observed frequencies in the TAM data. We then multiplied the differentiated cost per minute estimates by the observed duration of different actions and services at each clinic to estimate the cost per activity, cost per household visit, cost per day, and the total cost of the CCGs over a 12-month period. Costs are presented per CCG pair, unless otherwise specified.

#### Statistical analysis

We compared median values between the two clinics using quantile regression. Mean values between the clinics were calculated using linear regression with robust standard errors.

A detailed explanation of assumptions and calculations is available in the appendix. Data management, cleaning, and analysis were performed using STATA 11 (Stata Corp., College Station, TX, USA) and R Statistical Software v4.0.2 (R Foundation for Statistical Computing, Vienna, Austria).

#### Ethics approval and consent to participate

Oral informed consent was obtained from all participating CCG members before the commencement of data collection. This study was approved by the Human Research Ethics Committee (HREC) at the University of Witwatersrand and the London School of Hygiene & Tropical Medicine ethics committee. The described research adheres to the tenets of the Declaration of Helsinki.

## Results

Table 1 provides data on the characteristics of each clinic. Between March and October 2018, 159 self-reported daily TAM forms were included in the analysis out of 198 submitted from 11 CCG pairs (seven at clinic 1, four at clinic 2), totalling 39,667 minutes of data. CCG pairs spent a median time of 236 minutes (interquartile range [IQR]: 215, 261) per day conducting field activities at clinic 1, and a median 235 minutes (IQR: 201, 272) in the field at clinic 2. The shortest and longest days in the field lasted 105 and 345 minutes at clinic 1 and 114 and 325 minutes at clinic 2, respectively. CCG pairs at clinic 1 spent 49.5% (range: 40.3% - 53.4%) of their time directly interacting with patients at their households, compared to 35.0% (range: 33.0% - 38.1%) at clinic 2. It took a median of 8 minutes (IQR: 5, 15) for clinic 1 CCG pairs to travel between households and the clinic, compared to 14 minutes (IQR: 8, 20) for clinic 2 pairs (difference: 6 minutes, 95% Confidence Interval (CI): 5.1–6.9).

**Table 1:** Clinic characteristics.

Category	Sub-Category	Variable	Clinic One	Clinic Two
**General CCG Operational Characteristics**	General CCG Operational Characteristics	Rural/Urban	Peri-Urban	Urban
		Service Catchment Area (Sq. Km.)	3.1	0.6
		Annual Number of Households Served	8,035	5,200
		Number of on-site supervisors	2	1
		Number of CCG team members	15	9
		Number of CCG Pairs	7	4
**Operational Statistics Assessed via Time and Motion**	Time and Motion Study Summary	Total Number of TAM Days Reported	120	78
		Total Number of TAM Days Used in Analysis	101	58
		Average Number of TAM Days Reported per CCG Pair (Range)	14.4 (8, 18)	14.5 (12, 20)
		Total Person-Time Recorded Through TAM, Minutes	23,786	15,881
		Total Household Visit Time, Minutes	11,772	5,558
		Percent of Observation Time Spent at Households (Range)	49.5% (40.3, 53.4)	35.0% (33.0, 38.1)
		Median Person-Time Per Day per CCG Pair, Minutes (IQR)	236 (215, 261)	235 (201, 272)
	Households	Total Number of Attempted Household Visits	973	579
		Total Number of Successful Household Visits (%)	947 (97.3%)	414 (71.5%)
		Average Number of Households per Day	9.5	6.7
		Average Duration per Household Visit, Minutes (IQR)	11 (7, 15)	13 (9, 18)
	Unsuccessful Visits	Total Number of Unsuccessful Visits (%)	26 (2.7%)	165 (28.5%)
		Average Number of Unsuccessful Household Visits per Day	0.3	2.1
	Patients	Total Number of Patients	1756	460
		Average Number of Patients per Day	17.4	8.8
		Average Person-Time per Patient, Minutes	6.7	12.1
	Activities	Total Number of Household (non-travel) Activities Reported	1,692	391
		Percent of household visits specifically for tuberculosis care (Range)	3.9% (0, 21.1)	3.2% (0, 6.5)
		Percent of household visits specifically for non-tuberculosis care (Range)	43.9% (9.0, 100)	94.1% (87.0, 100)
		Percent of household visits with both tuberculosis and non-tuberculosis care (Range)	52.2% (0, 89.7)	2.7% (0, 6.5)
	Travel	Median Duration of Travel Time Between Households, Minutes (IQR)	8 (5, 15)	14 (8, 20)

CCG: Community Caregivers

CHW: Community Health Worker

IQR: Inter-Quartile Range

A total of 1,552 household visits and 2,083 household activities were recorded. CCG pairs at clinic 1 visited an average of 17.4 patients across 9.5 households per day (1.8 patients per household on average), spending a median of 11 minutes (IQR: 7, 15) at each house (6.7 minutes with each patient) (Table 1). At clinic 2, each CCG pair visited an average of 8.8 patients across 6.7 households per day (1.2 patients per household), spending a median of 13 (IQR: 9, 18) minutes at each household (12.1 minutes with each patient). CCG pairs at clinic 1 therefore visited an estimated 8.7 patients (95% CI: 8.4–9.1) and 2.8 households (95% CI: 2.2–3.4) more per day than CCG pairs in Clinic 2. Among successful household visits, 3.9% (range: 0, 21.1) at clinic 1 and 3.2% (range: 0, 6.5) at clinic 2 were exclusively for TB investigations, and 52.2% (range: 0, 89.7) of visits at clinic 1 and 2.7% (range: 0, 6.5) at clinic 2 involved some TB activities, but were not exclusive to TB care.

For both clinics, the most common activity performed at household visits were non-tuberculosis client encounters for screening/counselling/monitoring, taking 22.1% of total field time for clinic 1-based pairs and 25.5% for clinic 2-based pairs (Table 2).

**Table 2:** Frequency, median duration, and total duration of activity type by clinic.

	Clinic One				Clinic Two			
Activity Type^a^	Total Count of Activity Episodes Observed During TAM (% of total)	Total Person-Minutes Observed in TAM (%)	Mean Duration (95% Confidence Interval)	Median Duration (IQR)	Total Count of Activity Observed During TAM (% of total)	Total Person-Minutes Observed in TAM (%)	Mean Duration (95% Confidence Interval)	Median Duration (IQR)
**Adherence Support, Tuberculosis** ^b^	51 (1.8%)	451 (1.9%)	8.8 (6.1, 11.6)	5 (5, 10)	5 (0.5%)	45 (0.3%)	9.0 (3.0, 15.0)	8 (7, 11)
**Adherence Support, Other Disease** ^b^	386 (14.0%)	2633 (11.1%)	6.8 (6.2, 7.5)	5 (3, 8)	97 (9.4%)	1172 (7.4%)	12.1 (10.9, 13.3)	11 (8, 15)
**Client Encounter, Tuberculosis** ^c^	501 (18.1%)	3380 (14.2%)	6.8 (6.3, 7.2)	5 (3, 9)	17 (1.7%)	288 (1.8%)	16.9 (12.9, 21.0)	16 (11, 22)
**Client Encounter, Other Disease** ^c^	750 (27.1%)	5246 (22.1%)	7.0 (6.6, 7.4)	5 (3, 10)	272 (26.5%)	4053 (25.5%)	14.9 (13.8, 16.0)	14 (10, 18.5)
**Home-Based Care** ^d^	4 (0.1%)	62 (0.3%)	15.5 (-12.8, 43.8)	8 (5.5, 25.5)	0 (0%)	0 (0%)	0 (0, 0)	0 (0, 0)
**Travel**	1073 (38.8%)	12014 (50.5%)	11.2 (10.6, 11.8)	8 (5, 15)	637 (62.0%)	10323 (65.0%)	16.2 (15.3, 17.1)	14 (8, 20)
**Total**	2765	23786	--	--	1028	15881	--	--

^a^ See Table S1.1 in S1 Appendix for detailed explanation of each activity type.

^b^ Adherence Support: Stratified by whether visits were for Tuberculosis treatment or treatment for other diseases (e.g. HIV, hypertension, and diabetes). Visits by CCG pairs to households for Directly Observed Treatment (DOT), adherence counseling, medicine delivery or other forms of support for disease treatment.

^c^ Client Encounter: Stratified by whether visits were for tuberculosis treatment or treatment for other diseases. Examples of client encounter activities include general health screening, symptom screening (TB), sputum collection (TB), general health counseling and promotion, and contact tracing of households and household members.

^d^ Home-Based Care: All types of non-clinical, home-based care. Examples could include assistance with meal preparation, bathing, and similar activities.

TAM: Time and Motion

IQR: Inter-Quartile Range

Using a bottom-up costing approach, the total annual cost of CCG operations was estimated as $71,780 at clinic 1 and $49,097 at clinic 2. Per CCG pair, activities at clinic 1 incurred an average cost of $10,254, or $33.69 per CCG pair-day and $3.58 per successful household visit (Table 3, Tables S3.2. and S3.3 in S1 Appendix). Corresponding estimates in clinic 2 were $12,274 per CCG pair, $41.52 per CCG pair-day, and $5.85 per household successfully visited.

**Table 3:** Bottom-up cost estimates.

Category	Total Cost, Overall	Clinic One (7 CCG Pairs)				Clinic Two (4 CCG Pairs)			
		Total Cost	Daily Cost	Daily Cost of Field Activities^a^	Annual Cost per CCG Pair	Total Cost	Daily Cost	Daily Cost of Field Activities^a^	Annual Cost Per CCG Pair
**Equipment**	$4,646	$2,880	$11.52	$5.64	$411	$1,766	$7.54	$3.46	$442
**Staff**	$92,445	$54,943	$219.77	$107.60	$7,849	$37,502	$150.00	$73.44	$9,375
**Consumables**	$6,241	$3,972	$15.89	$7.78	$567	$2,270	$9.08	$4.44	$567
**Overhead**	$12,930	$7,496	$29.98	$14.68	$1,071	$5,434	$21.92	$10.64	$1,358
**Implementation**	$4,550	$2,457	$9.83	$4.81	$351	$2,093	$9.68	$4.10	$523
**Building**	$64.91	$32.46	$0.13	$0.06	$4.64	$32.46	$0.15	$0.06	$8.11
**Total**	$120,877	$71,780	$287.12	$140.57	$10,254	$49,097	$198.37	$96.14	$12,273

^a^ Assumes 250 working days a year, 8 hours a day. It was assumed that field activities (household visits and travel) took up 235 minutes per day on average, based on empiric observations, with the rest of the day spent at the main clinic/office.

CCG: Community Caregiver

**Fig 1:**
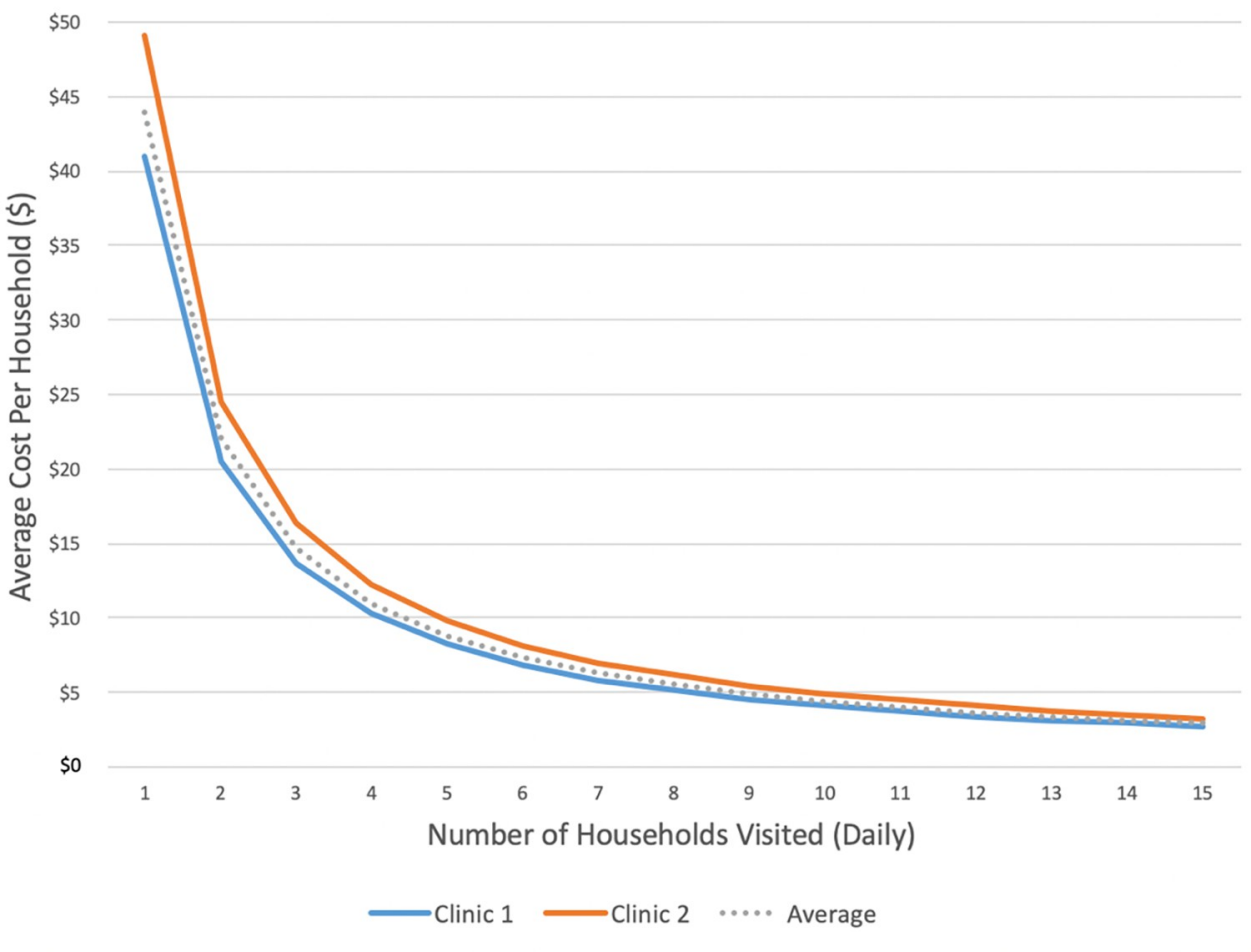
Average cost per household based on the number of households visited per day. Cost estimates are derived from the top-down cost estimates. As the number of households visited per days increases, the average cost per household decreases. Clinic Two has a more expensive cost per household at all values, but the difference in the cost per household between the clinics converges as the number of households visited per day increases.

Using a top-down approach, the average cost per CCG pair-day was $41.02 at clinic 1 and $49.10 at clinic 2. The average cost per household successfully visited was $4.37 (range: $4.15-$4.66) at clinic 1 and $6.88 (range: $5.93-$8.66) at clinic 2 (Tables S4.1 and S4.2 in S1 Appendix). The largest determinant of unit cost was the number of households visited, with the average cost per household visit decreasing as the number of household visits per day rose (Fig 1).

Over the full observation period, there were 26 unsuccessful visits and 947 successful visits at clinic 1, a 97.3% success rate (Tables 1 and 4). Clinic 2 had 165 unsuccessful visits and 414 successful visits, for a success rate of 71.5%. The most common reasons for an unsuccessful household visit were the target patient being unavailable or having the wrong address.

**Table 4:** Frequency of unsuccessful visits.

Reason for Unsuccessful Household Visit	Clinic One (N = 973 visit attempts)		Clinic Two (N = 579 visit attempts)	
	Frequency (%)	Average Number of Unsuccessful Visits per Month	Frequency (%)	Average Number of Unsuccessful Visits per Month
Patient Unavailable	18 (1.9%)	3.74	89 (15.4%)	24.5
Wrong Address	2 (0.2%)	0.42	43 (7.4%)	11.8
Deceased	3 (0.3%)	0.62	0 (0%)	0
Relocated	1 (0.1%)	0.21	1 (0.2%)	0.27
Door Locked	0 (0%)	0	10 (1.7%)	2.75
Refused Care	0 (0%)	0	1 (0.2%)	0.27
Unknown Patient	2 (0.2%)	0.42	21 (3.6%)	5.8
**Total**	**26 (2.7%)**	**5.4**	**165 (28.5%)**	**45.3**

## Discussion

Our study included over 1500 household visits made by 11 CCG pairs in Ekurhuleni District of Gauteng Province, South Africa. We report that CCG pairs perform a substantial amount of activities–thereby reducing burden to patients in seeking healthcare–at a modest cost to the healthcare system. Specifically, CCG pairs at both clinics were able to successfully complete seven to ten household visits per day, at a cost of $3-$6 per household successfully visited ($10,000-$12,500 per CCG pair per year). Our study also revealed variability in the number of successful household visits per day and the cost per household visit, driven in part by the higher frequency of unsuccessful visits in a more densely crowded, informally settled location. CCG teams were able to synergize TB contact investigations with other household health activities, with over one-third of all household visits involving both TB and non-TB evaluations.

At both sites, CCG pairs spent an average of about four hours per day in the field for household visits—with a large proportion of this time (greater than 50% at both sites) spent traveling between households, a finding consistent with an earlier study in Sedibeng and Umzinyathi districts in South Africa [21]. Despite spending a similar amount of time conducting household visits, the CCG pairs at clinic 1 (peri-urban) visited more households per day, spent less time at each household, and saw on average twice as many patients. There are several possible reasons for inter-clinic variability in the proportion of successful household visits, including both clinic-specific and external factors. First, the overall wealth and socioeconomic status of each catchment area may affect the likelihood of success, depending on the accessibility of household members for communications (e.g. by cell phone), ability to locate houses, and engagement of household members in out-of-household work. For example, in clinic 2’s catchment area, although households are generally close together, there are no formal street addresses. This leads to challenges in locating households and increases the travel time required between households, even though households are much more closely spaced than in clinic 1’s catchment area (a more formally settled, affluent area). The CCG pairs for clinic 2 therefore had more unsuccessful visits and greater travel time, despite having fewer pairs and making fewer attempted household visits during the observation period. These navigation challenges can also help explain why median travel time was shorter at clinic 1 (8 minutes) compared to clinic 2 (14 minutes). CCGs in similar settings could optimize their efficiency by planning travel routes to avoid unnecessary transit times between homes and instituting processes to reduce the frequency of unsuccessful household visits (e.g. closer pre-visit communication with households and more flexible scheduling options).

It is important for program and clinic managers to understand these challenges and the variability in success for CCGs across settings. If management is not aware of these issues, they could be prone to selecting strategies and approaches that are not cost-effective or do not align with the available resources (human and financial), resulting in inefficient or ineffective systems. These considerations, however, must be balanced against the possibility of greater need for accessible healthcare in regions like that served by clinic 2. Future research should combine estimates of cost and efficiency (as done here) with estimates of effectiveness, in terms of health outcomes.

The daily average cost per CCG pair was lower at clinic 1 than at clinic 2, despite clinic 1 having a larger total operating cost for its CCG program. This can be explained by economies of scale: clinic 1 had more CCG pairs, so fixed costs were distributed over a larger number of individuals, leading to a decreased marginal cost per additional CCG pair. The largest costs at both clinics were staff salaries and overhead expenses, and the biggest driver of unit cost was the number of household visits that each CCG pair was able to make in a day. The difference in costs between clinics demonstrates an important operational consideration: as more households are visited, the average cost per household decreases. And, as the number of household visits per day increased, the difference in cost per household between the two clinics decreased (i.e. there are diminishing marginal returns in savings). This is captured in the difference in operating costs between the two clinics evaluated in this study: a clinic that performs 9.5 household visits per day would spend $3.58 per household visit, whereas just making a few less visits each day (6.7 visits per day) would increase the cost to $5.85 per household visit.

CCG programs are not meant to replace clinic operations. Households and individual household members may choose to attend clinics directly, and all individuals can access public clinics and receive free treatment for diseases such as TB. But, there are still costs to patients for this care-seeking, such as travel time and lost wages. And South Africa does not have a national health insurance scheme at present, so for most individuals, these costs and any other costs of care are borne by individuals directly. Thus, CCGs could be, in terms of total cost, a more efficient alternative for patients.

While there are limited studies looking at the costs of community outreach interventions, one similar study investigated operations and costs of CCGs in deep-rural and peri-urban communities in South Africa [21]. While cost estimates are not directly comparable to those in this study (due to differences in methods and units of analysis), both studies highlight the importance of considering differentiated resource allocation that is contextualized to differences in supervision/management structure, geographical and demographic conditions, and healthcare needs in the communities where CCG teams operate. Another study by Lebina and colleagues (2020) looked at the cost of CCG (formerly WBOT) services as part of a larger integration package [22]. They also found that the cost of operating the program varied greatly depending on the size of the clinic, ranging from $145,228 for a clinic with 50 CHWs to $11,618 for a clinic with 4 CHWs—a cost comparable to that observed in our similarly sized clinics (15 and 9 CHWs for Clinic 1 and Clinic 2, respectively). Both studies also highlight the need to adequately fund and support CCG teams, considering the important role they serve in PHC. For example, CCG teams provide important infrastructure which can be utilized for effective and cost-effective contact tracing operations in low-resource settings. Contact tracing serves an important role in identification, monitoring, and treatment for many diseases, such as tuberculosis and COVID-19. But synergies from new service integration cannot be expected when CCG teams already face considerable workloads and resource constraints.

As with any study, our research has certain limitations. First, our study was only conducted at two clinics and we can draw only limited statistically significant conclusions with our clinic sample size. This limitation was the direct result of our voluntary participation study design (in that sites were provided the option to opt into, or out of, the costing analysis). However, the two clinics where data collection did occur represented distinct populations and social contexts, allowing us to highlight and contrast the operational challenges and operating costs between settings and set upper and lower bounds on the likely costs of CCG teams in other South African clinics. The use of two clinics also limited our ability to vary team composition within and across sites. Individuals composing each CCG pairing did rotate within each clinic, so that workers with differing levels of experience and work styles would spend time in the field together at various points during data collection. However, we could not randomly allocate CCGs across the two sites. We have made our data openly available on Github, should others wish to utilize our data in an expanded analysis of costs and operational requirements for CCGs in South Africa.

A second limitation was that time and motion data were self-reported, with the potential for bias and variability in the quantity and quality of data submissions over the study period. To enhance data quality, we provided routine feedback to CCG pairs on form submissions and developed a rating system to evaluate the quality of submitted forms, excluding those of the poorest quality. After review, more than 80% of submitted forms were considered of sufficient quality for inclusion in the analysis. Future studies can consider alternate ways of assessing time use for different commitments, such as time stamping data collection forms, automating logging systems, or direct observation of CCG activities by researchers [17]. Third, while the difference in per-visit costs between the sites was substantial, we do not have the ability—in the context of a study of two clinical trials sites—to disentangle the specific contextual factors that contributed to this difference, beyond the items (e.g. distance between clinics, frequency of unsuccessful visits) described above. Fourth, our data were limited to the evaluation of TB versus non-TB illness. Future research from a broader health system perspective could benefit from more detailed data on the specific non-TB illnesses evaluated.

## Conclusion

Our study provides insight into the human resources required to maintain CCG services for general health promotion, a comparison of feasibility and efficiency in different settings, and cost estimates for CCG care. The CCG framework offers important infrastructure to expand contact tracing efforts for several diseases, including TB and Covid-19. This study can be helpful to future managers as they consider implementation of CCGs in different settings. One of the biggest challenges with implementation of CCGs across South Africa has been confusion about the duties of CCGs, and the financial needs necessary to operate such a program. The findings from this study can help managers appropriately plan resource allocation for CCGs, understand the likely drivers of human resource needs for CCG implementation, and raise awareness about the need to carefully consider local contextual and operational factors (i.e. infrastructure of surrounding locale, workloads of potential CCG team members, and the scale of implementation) to ensure efficient and optimized implementation and operation of these programs.

## Supporting information

S1 Appendix (DOCX)
