# Supplementary material for: Human resource time commitments and associated costs of Community Caregiver outreach team operations in South Africa
Source: PLoS One. 2023 Mar 6;18(3):e0282425. doi: 10.1371/journal.pone.0282425 (PMC9987772; doi:10.1371/journal.pone.0282425)
Supplement: S1 Appendix — (DOCX) [file pone.0282425.s001.docx]

**Appendix 1. Additional Time-and-Motion Data**

**Table S1.1.** List of pre-defined activity codes and detailed description of each activity reported for the CCG time and motion study.

| **TAM Form** | **Type** | **Description** | **Activities included** |
| --- | --- | --- | --- |
| Daily Time-and-Motion Form | Visit Type | Household follow-up | Refer patients to clinic for antenatal and postnatal support, baby wellness and immunisation, health education, HIV counselling and education, deliver medication, provide support and monitor treatment adherence. |
|  |  | Household registration | Screen household for multiple health concerns, 1-on-1 patient interactions, counselling, delivering drugs, etc. |
|  |  | Trace other disease | Searching and calling (e.g. patient tracing) patients who have missed clinical and household visit appointments as well as other clinical follow-ups. |
|  |  | Trace Tuberculosis | Contact tracing for TB initiated by index case in the household, screening (clinical) for tuberculosis, sputum collection. |
|  | Travel | Travel | Time taken for travel (from clinic to household, household to another household and from household back to the clinic). |
| Household Visit TAM | Activities Performed | Client encounter for Tuberculosis | TB symptom screening, TB sample (sputum collection), education/counselling for TB, tracing non-adherent TB patients/treatment support, etc. |
|  |  | Client encounter for other disease | Other than TB: health screening, monitoring, promotion/education/counselling. |
|  |  | Adherence support Tuberculosis | TB drug delivery/treatment support, adherence counselling/support. |
|  |  | Adherence support other | Other than TB: drug delivery/treatment support, adherence counselling/support. |
|  |  | Home-based care | All types of home-based care, non-clinical (bathing, meal preparation, etc.). |

**Supplementary Table S1.2**. **Detailed Daily Time-and-Motion Data**

| **Pair Number** | **Clinic** | **Total Minutes** | **Total Direct Minutes (%)** | **Median Person-Time Per Day, min (IQR)** | **Median Travel Time Between Households, min (IQR)** | **Average Number of Activities Performed per Day** | **Average Number of Unsuccessful Household Visits per Day** |
| --- | --- | --- | --- | --- | --- | --- | --- |
| **1** | One | 4618 | 2353 (51.0%) | 261 (245, 276) | 9 (5, 15) | 17.78 | 0.33 |
| **2** | One | 3559 | 1733 (48.7%) | 240 (213, 278) | 10 (5, 15) | 16.4 | 0.13 |
| **3** | One | 3901 | 1910 (49.0%) | 243 (219, 263) | 7 (5, 15) | 18.31 | 0.69 |
| **4** | One | 3760 | 1898 (50.5%) | 228 (213, 251) | 6 (3.5, 12) | 17.81 | 0.13 |
| **5** | One | 3364 | 1751 (52.1%) | 234 (197, 251) | 9 (5, 14) | 11.53 | 0.27 |
| **6** | One | 2884 | 1469 (50.9%) | 225 (217, 240) | 6 (3, 13) | 20.85 | 0.00 |
| **7** | One | 1872 | 830 (44.3%) | 225 (198, 264) | 7.5 (5, 15.5) | 12.5 | 0.13 |
| **8** | Two | 4493 | 1781 (39.6%) | 235 (221, 254) | 16 (10, 29) | 4.11 | 2.16 |
| **9** | Two | 3751 | 1747 (46.6%) | 200 (183, 255) | 15 (11, 20) | 3.44 | 1.94 |
| **10** | Two | 6508 | 2803 (43.1%) | 270 (214, 285) | 13 (7, 20) | 5.85 | 2.85 |
| **11** | Two | 3198 | 1296 (40.5%) | 220 (210, 255) | 10 (7, 17) | 7.07 | 1.07 |
| **Total** | | 41908 | 19571 (46.7%) | 235 (210, 269) | 10 (5, 17) | 11.68 | 1.07 |
| **Clinic One** | | 23958 | 11944 (49.9%) | 236 (215, 261) | 8 (5, 15) | 16.75 | 0.26 |
| **Clinic Two** | | 17950 | 7627 (42.5%) | 235 (201, 275) | 14 (8, 20) | 5.08 | 2.14 |

**Supplementary Table S1.3**. **Detailed Data on Number of Patients and Average Person-Time per Patient**

| **Pair Number** | **Clinic** | **Number of Days with TAM Data** | **Total Number Household Visits** | **Total Patients** | **Average Household Visits per Day** | **Average Patients Per Day** | **Average Patients per HH** | **Average Person-Time per Household Visit** | **Average Person-Time Patient** |
| --- | --- | --- | --- | --- | --- | --- | --- | --- | --- |
| **1** | One | 18 | 179 | 306 | 9.94 | 17.00 | 1.67 | 25.80 | 15.43 |
| **2** | One | 15 | 132 | 264 | 8.80 | 17.60 | 1.97 | 26.96 | 13.69 |
| **3** | One | 16 | 156 | 287 | 9.75 | 17.94 | 1.82 | 25.01 | 13.77 |
| **4** | One | 16 | 159 | 304 | 9.94 | 19.00 | 1.89 | 23.65 | 12.52 |
| **5** | One | 15 | 135 | 214 | 9.00 | 14.27 | 1.57 | 24.92 | 15.84 |
| **6** | One | 13 | 125 | 255 | 9.62 | 19.62 | 2.04 | 23.07 | 11.31 |
| **7** | One | 8 | 76 | 126 | 9.50 | 15.75 | 1.66 | 24.63 | 14.86 |
| **8** | Two | 19 | 76 | 86 | 4.00 | 4.53 | 1.12 | 59.12 | 52.93 |
| **9** | Two | 18 | 42 | 75 | 2.33 | 4.17 | 1.21 | 89.31 | 73.83 |
| **10** | Two | 26 | 122 | 175 | 4.69 | 6.73 | 1.26 | 53.34 | 42.37 |
| **11** | Two | 14 | 109 | 124 | 7.79 | 8.86 | 1.14 | 29.34 | 25.79 |
| **Total** | | 178 | 1311 | 2216 | 7.37 | 12.45 | 1.63 | 31.97 | 19.62 |
| **Clinic One** | | 101 | 962 | 1756 | 9.52 | 17.39 | 1.80 | 24.90 | 13.80 |
| **Clinic Two** | | 77 | 349 | 460 | 4.53 | 5.97 | 1.19 | 51.43 | 43.27 |

**Supplementary Table S1.4. Frequency of activities by CCG pair**

| **Pair Number** | **Clinic** | **Adherence Support, Tuberculosis** | **Adherence Support, Other Disease** | **Ratio of Other:TB Adherence Visits** | **Client Encounter, Tuberculosis** | **Client Encounter, Other Disease** | | **Ratio of Other:TB Client Encounter** | **Home-Based Care** |
| --- | --- | --- | --- | --- | --- | --- | --- | --- | --- |
| **1** | One | 2 | 61 | 30.5 | 126 | 131 | 1.0 | | 2 |
| **2** | One | 11 | 23 | 2.1 | 107 | 105 | 1.0 | | 0 |
| **3** | One | 1 | 43 | 42.0 | 140 | 110 | 0.8 | | 1 |
| **4** | One | 0 | 33 | 0 | 123 | 129 | 1.0 | | 0 |
| **5** | One | 3 | 77 | 25.7 | 3 | 90 | 30.0 | | 0 |
| **6** | One | 0 | 145 | 0 | 0 | 126 | 0 | | 0 |
| **7** | One | 34 | 5 | 0.2 | 2 | 59 | 29.5 | | 1 |
| **8** | Two | 0 | 13 | 0 | 0 | 65 | 0 | | 0 |
| **9** | Two | 0 | 4 | 0 | 0 | 58 | 0 | | 0 |
| **10** | Two | 2 | 49 | 24.5 | 16 | 85 | 5.3 | | 0 |
| **11** | Two | 3 | 31 | 10.3 | 1 | 64 | 64.0 | | 0 |
| **Total** | | 56 | 483 | 8.6 | 518 | 1022 | 2.0 | | 4 |
| **Clinic One** | | 51 | 386 | 7.6 | 501 | 750 | 1.5 | | 4 |
| **Clinic Two** | | 5 | 97 | 19.4 | 17 | 272 | 16.0 | | 0 |


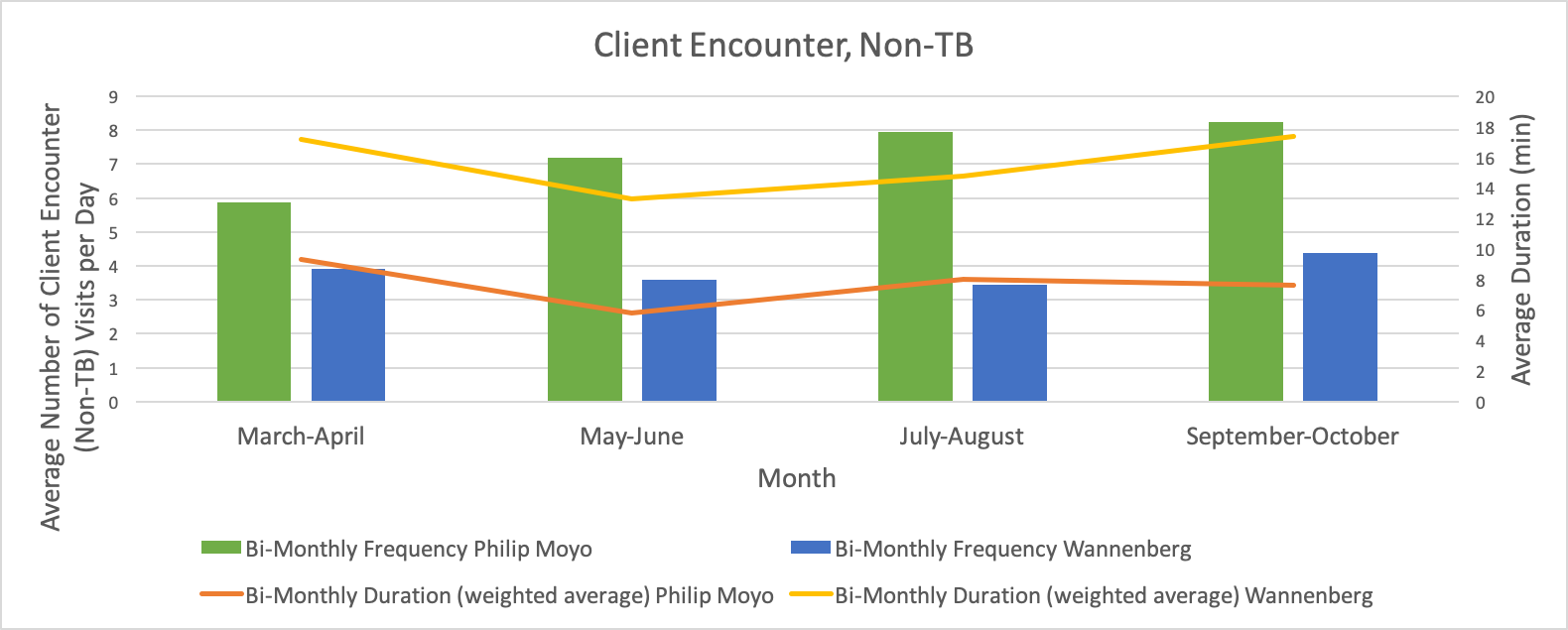

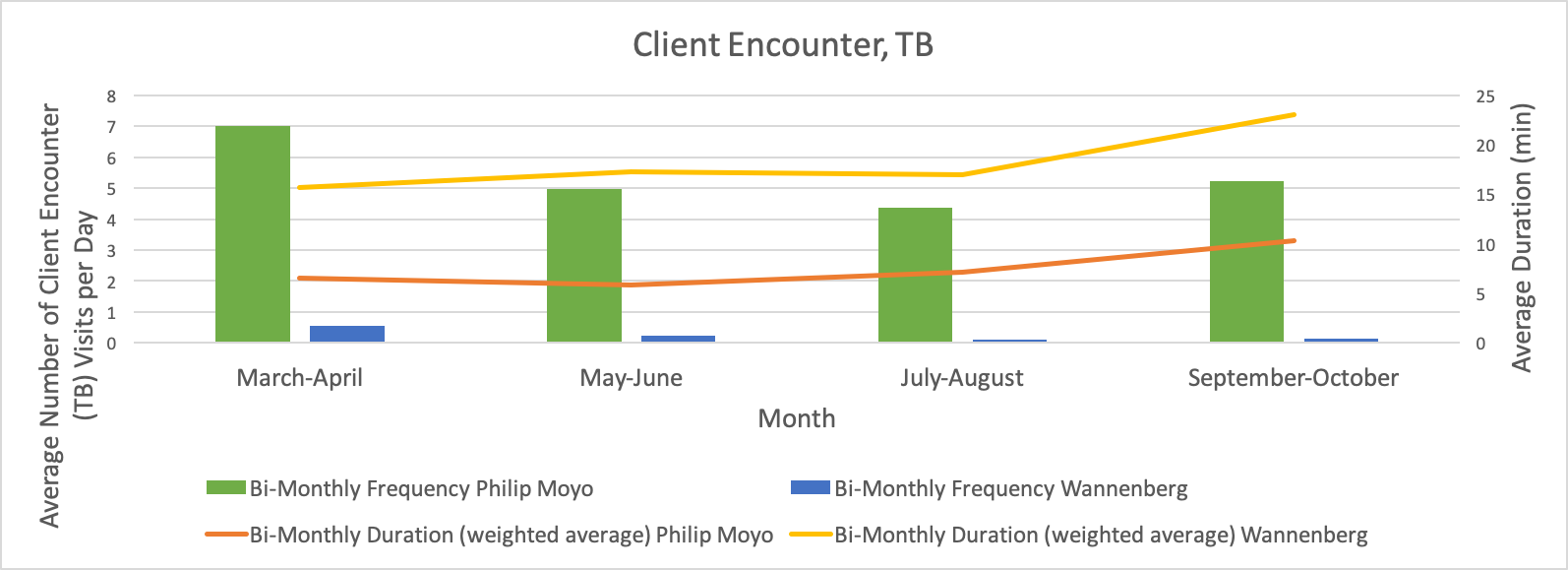


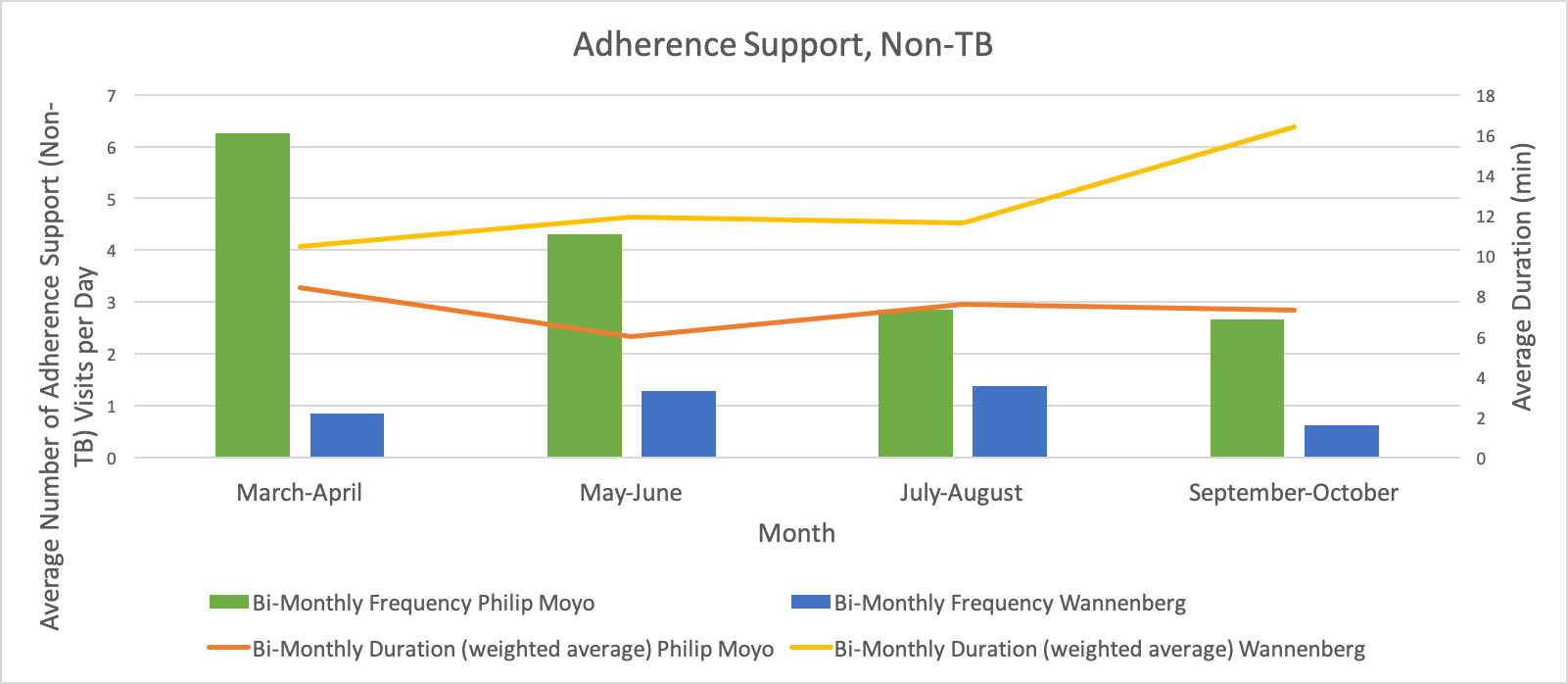

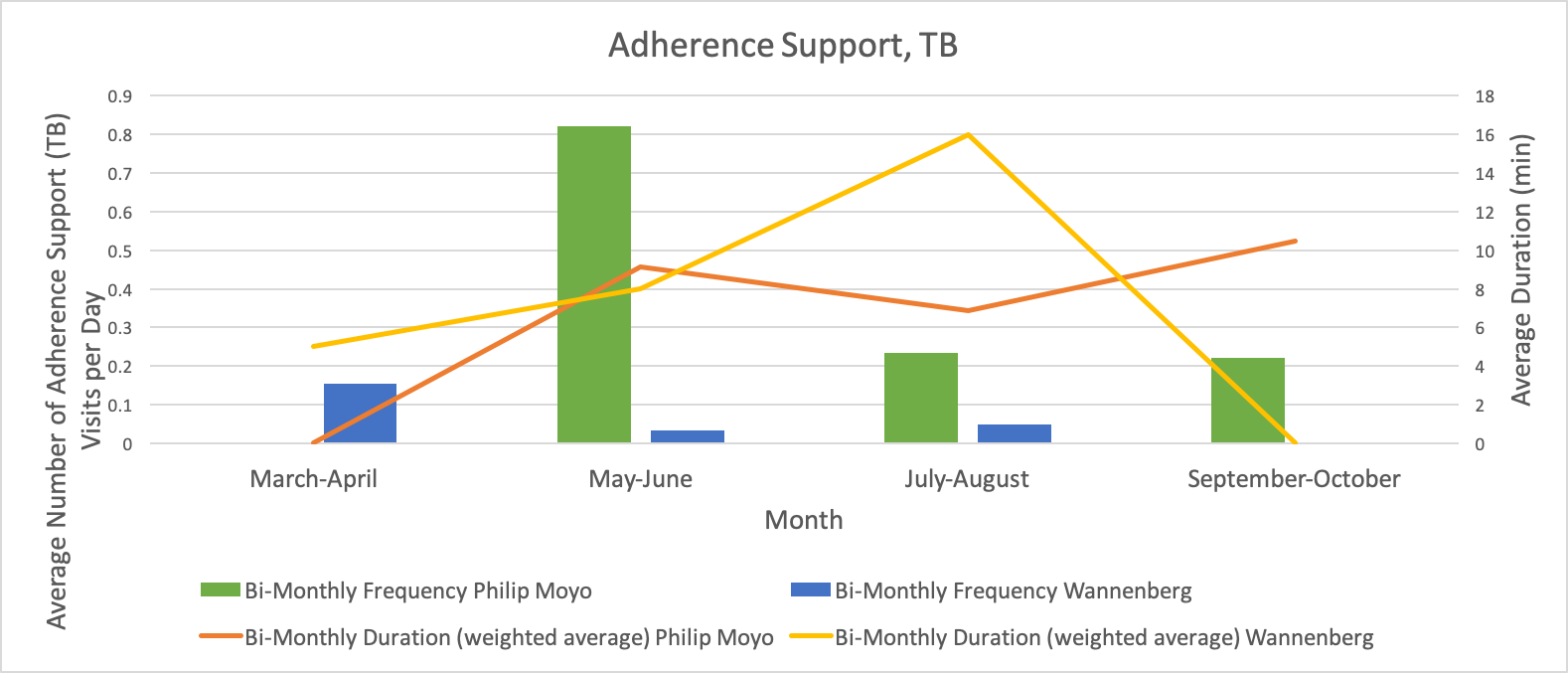


**Supplementary Figure S1.1**. Frequency and Average Duration of Household Activities Over Time (4-way panel)

Each panel corresponds to one of the major activities recorded by CCG pairs. Home-based care activities were excluded due to the small number of observations. The table depicts trends in the frequency and duration of each activity over 2-month periods throughout the study period. Columns represent the frequency per day each activity was performed during the study period, lines represent the average duration of each activity type. There were no major changes in the frequency or duration of household activities during the study period.

**Appendix 2: Detailed information on Unsuccessful Household Visits**

**Supplementary Table S2.1. Number of Unsuccessful Visits and Reason by Pair**

| **Pair Number** | **Clinic** | **Patient Unavailable** | **Wrong Address** | **Deceased** | **Relocated** | **Door Locked** | **Refused Care** | **Unknown Patient** | **Total** |
| --- | --- | --- | --- | --- | --- | --- | --- | --- | --- |
| **1** | One | 4 | 1 | 0 | 0 | 0 | 0 | 1 | 6 |
| **2** | One | 2 | 0 | 0 | 0 | 0 | 0 | 0 | 2 |
| **3** | One | 6 | 1 | 2 | 1 | 0 | 0 | 1 | 11 |
| **4** | One | 1 | 0 | 1 | 0 | 0 | 0 | 0 | 2 |
| **5** | One | 4 | 0 | 0 | 0 | 0 | 0 | 0 | 4 |
| **6** | One | 0 | 0 | 0 | 0 | 0 | 0 | 0 | 0 |
| **7** | One | 1 | 0 | 0 | 0 | 0 | 0 | 0 | 1 |
| **8** | Two | 12 | 17 | 0 | 0 | 2 | 1 | 9 | 41 |
| **9** | Two | 23 | 6 | 0 | 0 | 0 | 0 | 6 | 35 |
| **10** | Two | 42 | 20 | 0 | 0 | 6 | 0 | 6 | 74 |
| **11** | Two | 12 | 0 | 0 | 1 | 2 | 0 | 0 | 15 |
| **Total** | | 107 | 45 | 3 | 2 | 10 | 1 | 23 | 191 |
| **Clinic One** | | 18 | 2 | 3 | 1 | 0 | 0 | 2 | 26 |
| **Clinic Two** | | 89 | 43 | 0 | 1 | 10 | 1 | 21 | 165 |

**Appendix 3. Detailed Bottom-Up Results**

**Supplementary Table S3.1. Proportion of cost/min to each activity**

| **Proportion of cost/min to each activity** | | |
| --- | --- | --- |
| **Activity** | **Clinic One proportion** | **Clinic Two proportion** |
| Household Follow-Up | 0.38 | 0.21 |
| Household Registration | 0.06 | 0.17 |
| Trace Other | 0.04 | 0.08 |
| Trace Tuberculosis | 0.00 | 0.01 |
| Travel | 0.52 | 0.53 |
| Follow-Up + Registration | 0.00 | 0.01 |

**Supplementary Table S3.2. Total costs and cost per minute by arm**

|  | | **Clinic One** | | | | **Clinic Two** | | | |
| --- | --- | --- | --- | --- | --- | --- | --- | --- | --- |
| **Category** | **Total Cost, Overall** | **Total Cost** | **Cost per WBOT Pair** | **Total Cost Per Min** | **Cost Per Min Per WBOT Pair** | **Total Cost** | **Cost Per WBOT Pair** | **Total Cost Per Min** | **Cost Per Min Per WBOT Pair** |
| **Equipment** | $4,646 | $2,880 | $411 | $0.0240 | $0.0034 | $1,766 | $442 | $0.0147 | $0.0037 |
| **Staff** | $92,445 | $54,943 | $7,849 | $0.4579 | $0.0654 | $37,502 | $9,375 | $0.3125 | $0.0781 |
| **Consumables** | $6,241 | $3,972 | $567 | $0.0331 | $0.0047 | $2,270 | $567 | $0.0189 | $0.0047 |
| **Overhead** | $12,930 | $7,496 | $1,071 | $0.0625 | $0.0089 | $5,434 | $1,358 | $0.0453 | $0.0113 |
| **Implementation** | $4,550 | $2,457 | $351 | $0.0205 | $0.0029 | $2,093 | $523 | $0.0174 | $0.0044 |
| **Building** | $64.91 | $32.46 | $4.64 | $0.0003 | $0.0000 | $32.46 | $8.11 | $0.0003 | $0.0001 |
| **Travel** | $- | $- | $- | $- | $- | $- | $- | $- | $- |
| **Total** | $120,877 | $71,780 | $10,254 | $0.5982 | $0.0855 | $49,097 | $12,273 | $0.4091 | $0.1023 |

**Supplementary Table S3.3. Duration and Cost of Activities at Each Clinic**

|  | **Clinic One** | | | | **Clinic Two** | | | |
| --- | --- | --- | --- | --- | --- | --- | --- | --- |
| Activity | Average Frequency per Day | Cost per Minute | Cost per Activity | Total Cost per Day | Overall Frequency per Day | Cost per Minute | Cost per Activity | Total Cost per Day |
| Household Follow-Up | 7.69 | $0.15 | $1.83 | $14.11 | 3.35 | $0.18 | $2.01 | $6.74 |
| Household Registration | 1.20 | $0.14 | $1.50 | $1.79 | 2.62 | $0.18 | $2.83 | $7.41 |
| Household Follow-Up & Registration | 0 | $0.14 | -- | -- | 0.09 | $0.18 | $3.45 | $0.31 |
| Trace, Other Disease | 0.71 | $0.14 | $1.49 | $1.06 | 1.27 | $0.18 | $2.45 | $3.12 |
| Trace, Tuberculosis | 0.03 | $0.14 | $1.78 | $0.05 | 0.13 | $0.18 | $1.87 | $0.24 |
| Travel | 10.62 | $0.14 | $1.57 | $16.68 | 0.09 | $0.18 | $3.45 | $23.70 |
| Total Per Day | 20.26 | $0.14 | -- | $**33.69** | 15.74 | $0.18 | -- | $**41.52** |

**Appendix 4. Detailed Top-Down Results**

**Supplementary Table S4.1. Top-Down Cost Estimates, Cost per Pair and Cost per Community-Health Worker**

|  | **Facility** | **Cost Per Pair** | **Cost per Community Health Worker** |
| --- | --- | --- | --- |
| **Daily Cost** | Clinic One | $41.02 | $20.51 |
|  | Clinic Two | $49.10 | $24.55 |
| **Annual Cost** | Clinic One | $10,254 | $5,127 |
|  | Clinic Two | $12,274 | $6,137 |

**Supplementary Table S4.2. Top-Down Average Unit Costs, by CCG Pair**

| **Pair Number** | **Clinic** | **Average Number of Patients per Day** | **Average Cost per Patient** | **Average Number of Household Visits per Day** | **Average Cost per Household** |
| --- | --- | --- | --- | --- | --- |
| **1** | One | 17 | $2.41 | 9.8 | $4.17 |
| **2** | One | 17.6 | $2.33 | 8.8 | $4.66 |
| **3** | One | 17.9 | $2.29 | 9.3 | $4.43 |
| **4** | One | 19.0 | $2.16 | 9.9 | $4.15 |
| **5** | One | 14.3 | $2.87 | 8.9 | $4.63 |
| **6** | One | 19.6 | $2.09 | 9.5 | $4.30 |
| **7** | One | 15.8 | $2.60 | 9.4 | $4.38 |
| **8** | Two | 7.2 | $5.70 | 6.3 | $7.75 |
| **9** | Two | 6.3 | $6.51 | 5.7 | $8.66 |
| **10** | Two | 8.8 | $4.66 | 7.7 | $6.38 |
| **11** | Two | 8.9 | $4.61 | 8.3 | $5.93 |
| **Total** | | 13.9 | $2.95 | 8.6 | $5.14 |
| **Clinic One** | | 17.4 | $2.36 | 9.5 | $4.37 |
| **Clinic Two** | | 7.9 | $5.19 | 6.7 | $6.88 |

**Appendix 5. Detailed Top-Down and Bottom-Up Methodology**

**Bottom-Up Cost Estimates**

Costs for CCG in the Asibambisane study were broken up into 8 separate categories: training, capital (implementation), capital (existing infrastructure), service provision, supervision, overhead, consumables, and building/furniture.

Training costs and capital (implementation) costs were assessed as the implementation costs for the CCG intervention. These were costs incurred by the clinics in order to launch/initiate CCG operations at their clinics. Training costs consisted of expenses like renting out a venue, providing training manuals, and facilitator fees. It was assumed that refresher trainings would have to be conducted every 5 years, so costs were annuitized for a 5-year time period. Capital (implementation) refers to durable goods purchased as a part of the implementation/start-up. In Asibambisane, capital expenses related to intervention implementation included subscriptions to a special app used by CCGs at household visits, tablets for each CCG pair, computers for clinics, and insurance to cover all capital purchased (charged as 10% of cost for each item). The unit costs for all training and capital (implementation) expenses were charged per individual or per CCG pair, with the exception of computers (one per clinic).

Capital (existing infrastructure) and consumables refer to goods and materials already available at study sites that were used by CCGs to carry out household visits. Existing capital infrastructure consisted of multi-use items such as glucometers and blood pressure machines. These goods were annuitized based on their expected life-years, assuming each CCG pair would require all capital goods. Consumables refers to single-use items such as alcohol swabs, sanitary pads, and gloves. It was assumed that all CCG pairs would require 50 of each consumable good per month.

Building/furniture and overhead costs refer to operating expenses of each clinic. Building/furniture refers to expenses such as rent, floor space, and furniture such as desks and chairs that are used by CCGs while at the clinic. In the study, the CCGs did not use office space, but would sit in the clinic meeting rooms when completing admin tasks and prepping for the day. So, the cost of these meeting spaces was used to estimate building costs. Overhead costs refer to utilities such as electricity, water, and security, as well as administrative expenses for a central CCG coordinator whom is shared across all CCG sites. For this study, utility costs were estimated at 10% of total expenses incurred at each site. The CCG coordinator expense was split evenly across all clinics providing CCG services.

Last, the service provision and supervision costs were used to estimate the human resource/personnel costs to operate the CCG intervention. All salaries were taken from a public portal from the South African Ministry of Health.^1^ Service provision was the salary costs of the community health workers (CHW) that composed the CCG pairs. Supervision costs were the salaries of the CCG supervisors. There were two supervisors for the CCGs. Each clinic had a local outreach team leader, who would oversee the day-to-day CCG operations. Their salary was paid in-full by the clinic. In addition, there was a central manager, who oversaw the CCG operations for the entire district. The manager’s salary was split evenly across all clinics implementing the CCG intervention.

For all expenses, we estimated a total cost per minute by assuming 250 working days a year, working 8 hours per day. We then took the total cost for each of the categories described above and divided it by (250 days * 8 hours * 60 minutes). This per-minute estimate of cost was integrated with the time-and-motion data to estimate the cost of a typical routine visit, travel time, as well as the average CHW salary cost to complete different CCG activities at a household. We apportioned costs to different activities based on the frequency, duration, and total time dedicated to them. By constructing cost estimates for each of the different aspects of CCG activities, we were able to build estimates of the total cost per individual, per CCG pair, and per clinic. The average cost per day was estimated by calculating the average cost for each activity and the average frequency/number of times each activity was performed in a day, to estimate a total cost per activity. The total costs for each separate activity were then summed together to build an estimate of the total daily cost for a CCG pair.

**Top-Down Cost Estimates**

Using the estimates of total cost, aggregate top-down estimates were then constructed for several unit cost outcomes. First, the daily cost per CCG pair was estimated by taking the total operating cost at each clinic, dividing it by the number of annual operating days (assumed to be 250 days), and then dividing it by the number of CCG pairs working at each clinic. Once the daily cost per pair was estimated at each clinic, the cost per patient and individual was estimated separately for each pair. For this estimation, the daily cost per pair was divided by the average number of patients and the average number of households visited by each pair, based on the time and motion data. The cost per household was estimated as the average number of patients/households visited by CCG pairs per day, divided by the total daily cost at the clinic. Last, the graph of cost per household versus the number of households visited in a day (Figure 1) was estimated by taking the average cost per day for a CCG pair and dividing it by the number of households visited in a day, at each point on the x-axis.

1. Department of Public Service and Administration, Republic of South Africa [Internet]. Salary Scales, with Translation Keys, for Employees on Salary Levels 1 to 12 and Those Employees Covered by Occupation Specific Dispensations (OSD); 2018 [Cited December 5, 2019]. Available from: https://www.westerncape.gov.za/text/2018/July/2018_cost_of_living_adjustments.pdf.

**Appendix 6. Wealth Distribution of Catchment Area, by Clinic**

**Supplementary Table S6.1. Average household income (annual), Clinic 1 Catchment Area**

| **Income** | **Percentage** |
| --- | --- |
| No income | 18.7% |
| R1 - R4,800 | 5.6% |
| R4,801 - R9,600 | 8.7% |
| R9,601 - R19,600 | 16.7% |
| R19,601 - R38,200 | 22.1% |
| R38,201 - R76,400 | 16.6% |
| R76,401 - R153,800 | 7.7% |
| R153,801 - R307,600 | 2.9% |
| R307,601 - R614,400 | 0.9% |
| R614,001 - R1,228,800 | 0.1% |
| R1,228,801 - R2,457,600 | 0.1% |
| R2,457,601+ | 0% |

Source: Statistics South Africa. Statistics by place: Etwatwa. [Cited 11 November 2022] Available from: <https://www.statssa.gov.za/?page_id=4286&id=11269>.

**Supplementary Table S6.2. Average household income (annual), Clinic 2 Catchment Area**

| **Income** | **Percentage** |
| --- | --- |
| No income | 15.2% |
| R1 - R4,800 | 3.1% |
| R4,801 - R9,600 | 3.7% |
| R9,601 - R19,600 | 8.5% |
| R19,601 - R38,200 | 14.2% |
| R38,201 - R76,400 | 13.2% |
| R76,401 - R153,800 | 12.6% |
| R153,801 - R307,600 | 13% |
| R307,601 - R614,400 | 10.3% |
| R614,001 - R1,228,800 | 4.4% |
| R1,228,801 - R2,457,600 | 1.2% |
| R2,457,601+ | 0.6% |

Source: Statistics South Africa. Statistics by place: Germiston. [Cited 11 November 2022] Available from: <https://www.statssa.gov.za/?page_id=4286&id=11265>.

**
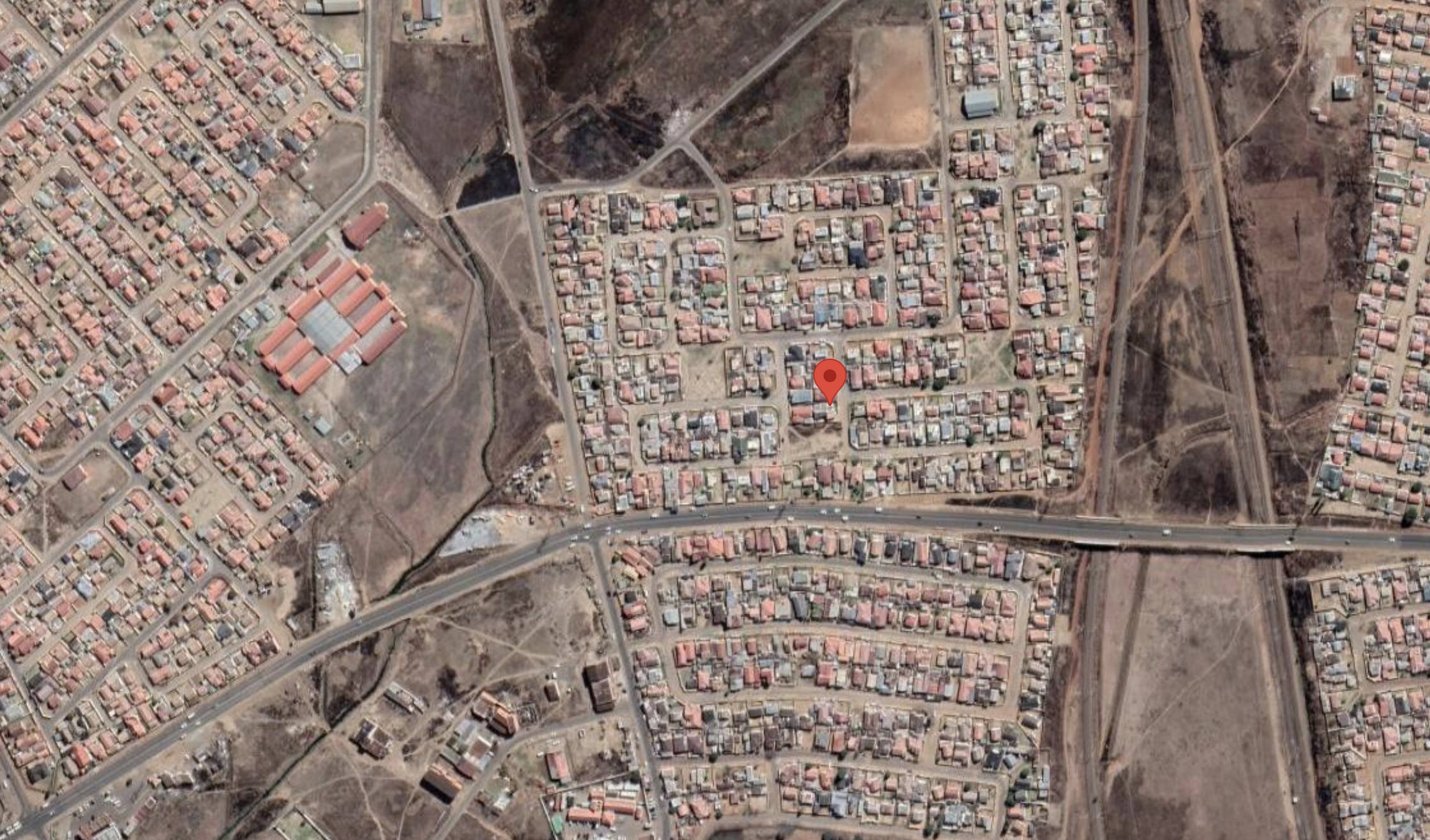
**

**Figure S6.1. Area Surrounding Clinic 1**

Source: Google Maps. [Area surrounding Clinic 1]. Accessed 11 November 2022.


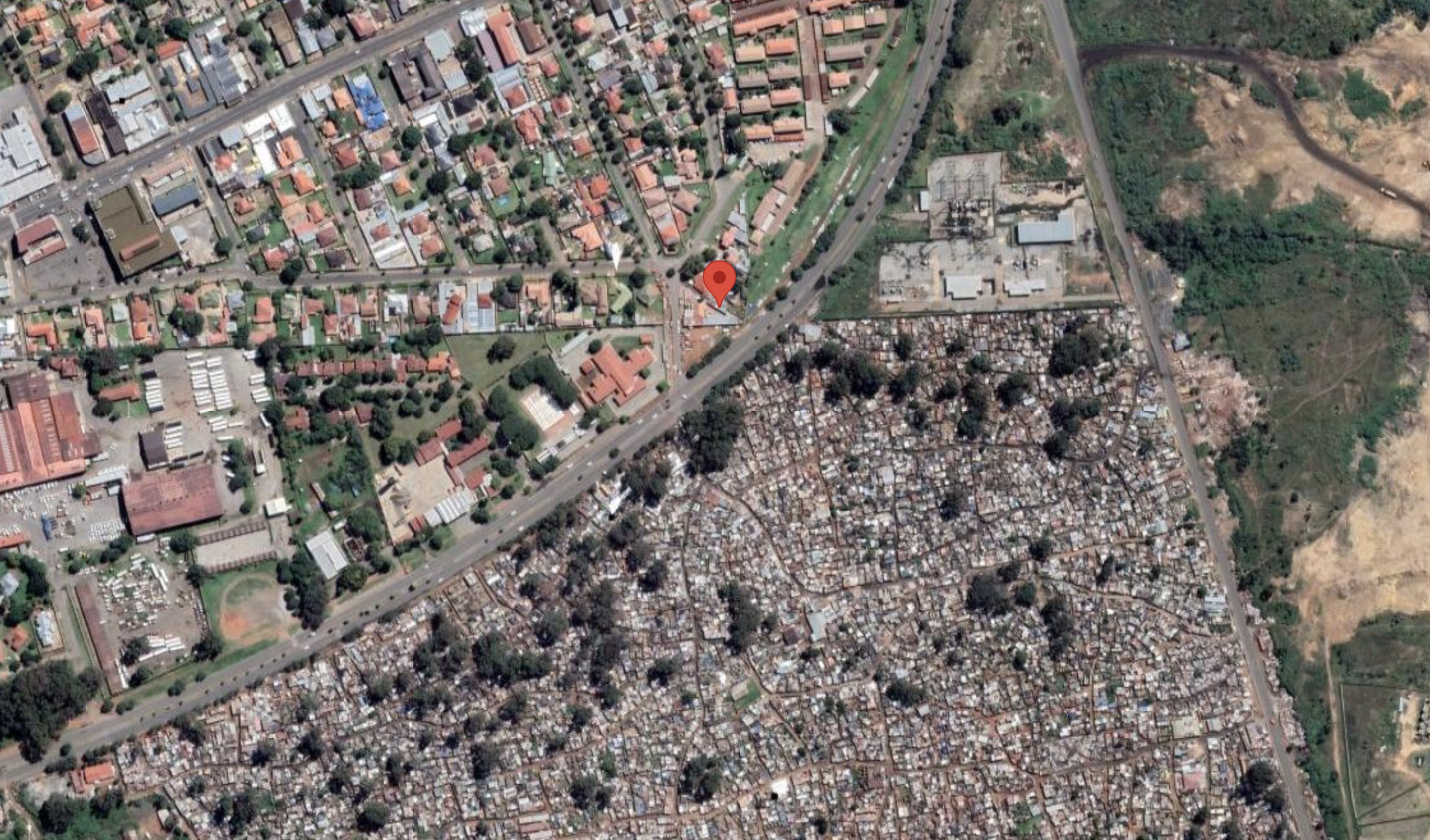


**Figure S6.2. Area Surrounding Clinic 2**

Source: Google Maps. [Area surrounding Clinic 2]. Accessed 11 November 2022.
